# Communication between cells: exosomes as a delivery system in prostate cancer

**DOI:** 10.1186/s12964-021-00792-1

**Published:** 2021-11-12

**Authors:** Pia Giovannelli, Marzia Di Donato, Giovanni Galasso, Alessandra Monaco, Fabrizio Licitra, Bruno Perillo, Antimo Migliaccio, Gabriella Castoria

**Affiliations:** 1 Dipartimento di Medicina di Precisione, Università Della Campania ‘L. Vanvitelli’, Via L. De Crecchio, 7, 80138 Naples, Italy; 2 Istituto di Scienze dell’Alimentazione, C.N.R., 83100 Avellino, Italy

**Keywords:** Prostate cancer, Tumor microenvironment, Exosomes, Diagnostic guidance, Therapeutic applications

## Abstract

Despite the considerable efforts in screening and diagnostic protocols, prostate cancer still represents the second leading cause of cancer-related death in men. Many patients with localized disease and low risk of recurrence have a favourable outcome. In a substantial proportion of patients, however, the disease progresses and becomes aggressive. The mechanisms that promote prostate cancer progression remain still debated. Many findings point to the role of cross-communication between prostate tumor cells and their surrounding microenvironment during the disease progression. Such a connection fosters survival, proliferation, angiogenesis, metastatic spreading and drug-resistance of prostate cancer. Recent years have seen a profound interest in understanding the way by which prostate cancer cells communicate with the surrounding cells in the microenvironment. In this regard, direct cell-to-cell contacts and soluble factors have been identified. Increasing evidence indicates that PC cells communicate with the surrounding cells through the release of extracellular vesicles, mainly the exosomes. By directly acting in stromal or prostate cancer epithelial cells, exosomes represent a critical intercellular communication system. By querying the public database (https://pubmed.ncbi.nlm.nih.gov) for the past 10 years, we have found more than four hundred papers. Among them, we have extrapolated the most relevant about the role of exosomes in prostate cancer malignancy and progression. Emerging data concerning the use of these vesicles in diagnostic management and therapeutic guidance of PC patients are also presented.

**Video Abstract**

**Supplementary Information:**

The online version contains supplementary material available at 10.1186/s12964-021-00792-1.

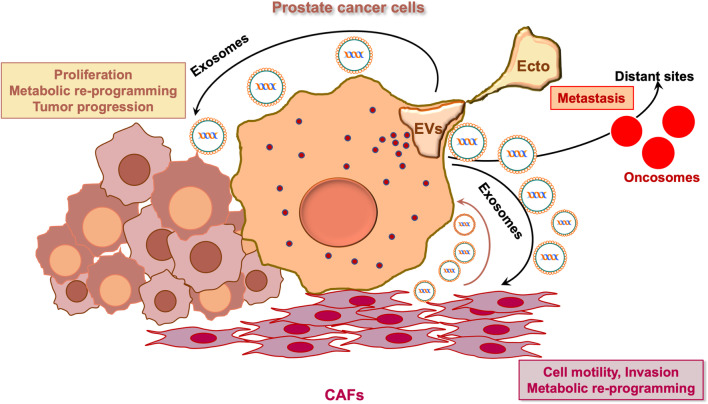

## Background

Prostate cancer (PC) remains the most commonly diagnosed non-cutaneous malignancy in men. The starting events in PC involve intrinsic changes in epithelial prostate cells, in combination with distinct changes in the surrounding microenvironment. The consequences of these interactions can be profound, ranging from cell survival to angiogenesis, metabolic reprogramming, resistance to therapy, escape from immune surveillance and metastatic spreading. These hallmarks lead to progression and development of metastatic disease, which is almost untreatable. Currently used therapies, indeed, initially show antitumor effect to become almost inefficacious as PC progresses [1]. As such, a better understanding of the mechanisms responsible for disease progression and drug-resistance represents a challenging puzzle.

PC cells undergo epithelial-mesenchymal transition (EMT) to drive metastasis in distant organs. This process enables a gain of functions, such as the loss of cell–cell contacts, a robust increase in proliferation and acquisition of a high-motile phenotype [2]. Once detached from the primary site, tumor cells invade extracellular matrix (ECM) and disseminate into the bloodstream and lymphatics, where they are detectable as circulating tumor cells (CTCs). These cells often survive to the journey through the circulation, and once reached the metastatic site, they might co-opt the resident stromal cells to generate a ‘permissive’ microenvironment. It has been consistently shown that PC cells alter the microenvironment to allow the development of metastases only when metastatic sites contain a cluster of specialized cells, known as metastases-initiating cells (MICs). These cells recruit and re-program prostate transformed and non-transformed epithelial as well as stromal cells to control metastatic events [3]. Although simple in their summarization, these processes require participation of lead actors, extras and ‘bystander’ cells.

Therefore, there is a great interest in understanding the way by which PC cells communicate with the surrounding stromal cells. A lot of papers have so far identified direct cell-to-cell contacts, soluble factors, nutrients and hormones implicated in this liaison [4–11]. Increasing evidence, however, indicates that extracellular vesicles (EVs), including the exosomes, mediate the complex cross talk between stroma and cancer epithelial cells [12].

Exosomes deliver a *plethora* of molecules. Therefore, they represent a critical intercellular communication system for exchange of information between cells. The lipid bilayer membrane of exosomes protects their cargo from RNases and proteases [13]. Thus, they may also act as an efficient delivery system in therapy. Exosomes mediate prostate carcinogenesis as well as PC progression by directly acting on cancer epithelial cells or stromal cells, or even by reprogramming the ‘dormant’ cells in tumor microenvironment [14]. Nevertheless, the role and molecular signatures of exosomes during PC progression and drug-resistance are still under investigation. Even less clear appears the role of exosome-derived androgen receptor (AR) in PC progression and hormone-resistance. The present manuscript aims to fill this gap.

## EVs: structure and functions

EVs are circular membrane-enclosed particles secreted by almost all cell types during their physiological and pathological processes. Cardiovascular, neuronal, immune and non-immune cells release EVs. Although their physiological role needs to be further elucidated in vivo, EVs contribute to pathogenesis of cardiovascular as well as neurodegenerative diseases and regulate the immuno-responses, including the host-response to viral infections. Cancer cells also release EVs [15].

EVs generally fall into two major categories, ectosomes and exosomes [16], although their classification is far to be defined [17]. Ectosomes are generated by the direct outward budding of plasma membrane, which produces micro-vesicles, micro-particles, and large vesicles in the size range of ~ 50 nm–1 μm in diameter. Exosomes, instead, are of endosomal origin and have a size range of ~ 40–160 nm in diameter. They derive from a process involving the formation of intracellular multivesicular bodies (MVBs), containing intraluminal vesicles (ILVs), which are secreted as exosomes through MVB fusion to plasma membrane and the subsequent exocytosis. These processes are finely regulated by effectors involved in the intracellular vesicular trafficking, such as the Ras-related GTPase, Rab or the endosomal sorting complexes required for transport (ESCRT) of proteins, phospholipids and ceramides.

**Fig. 1:**
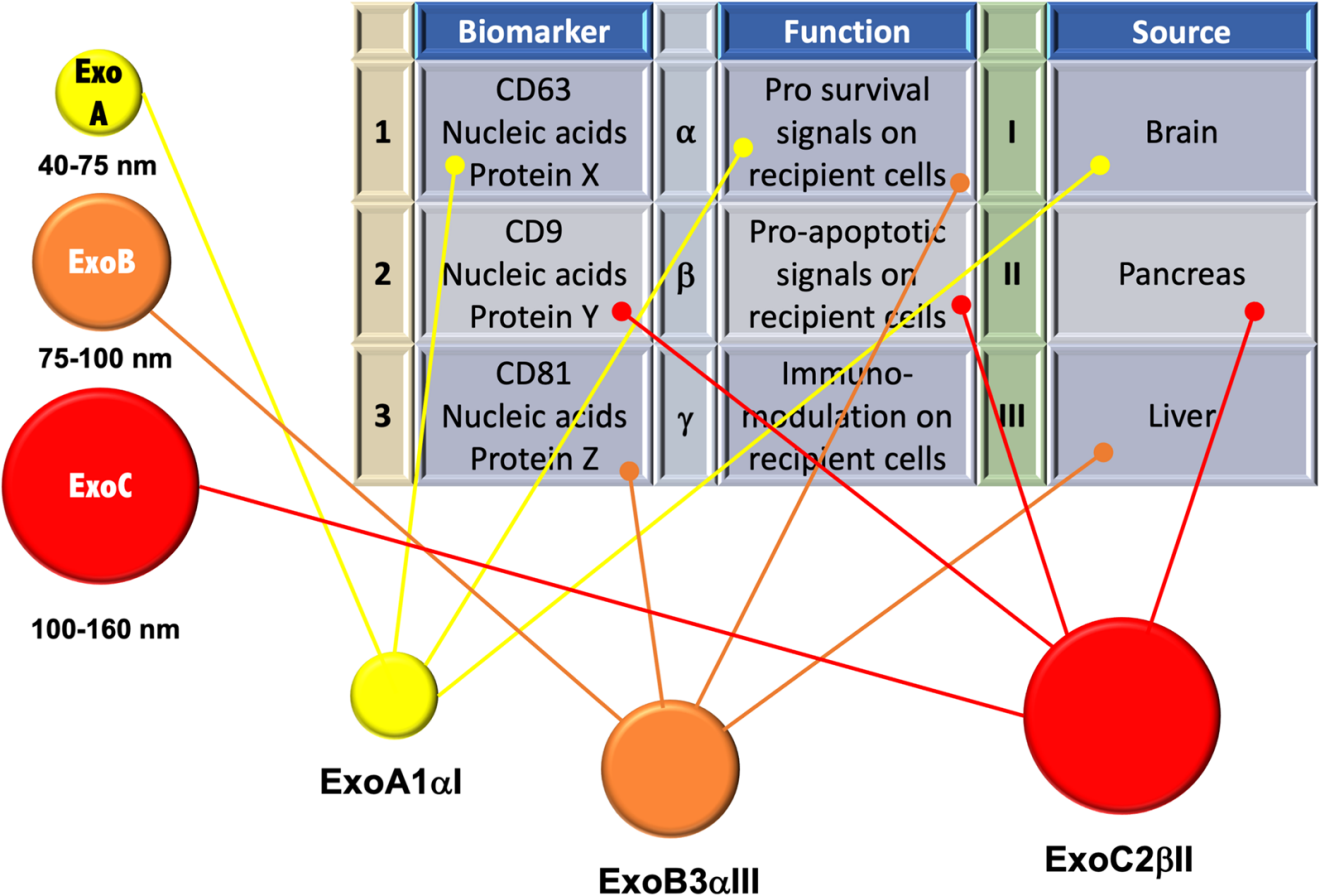
A schematic representation of the different exosomes grouped by size, contents, functions and localization is shown

Exosomes deliver cytoplasmic proteins, nucleic acids, lipids and glycol-conjugates protected by a lipid bilayer. Their composition often reflects the identity of the cell of origin, but at the same time exosomes exhibit some unique mixtures of genetic material [18]. Notably, exosomes show a great heterogeneity, which reflects their content, origin and size (Fig. 1). Cross-combination of these characteristics allows a great range of functions, including survival, immuno-response, and so on. The role of exosomes in recipient cells remains, however, an important challenge. They might directly stimulate acceptor cells by interaction with surface-expressed ligands or transfer membrane receptors between cells or mediate the horizontal transfer of proteins and genetic information, such as small or long non-coding RNAs, structural RNAs, tRNAs, and small interfering RNAs between cells [19–22]. When exosomes deliver oncogenic material that might induce transformation of the recipient cells, they are usually named oncosomes [23]. Multiple signaling pathways affected by tumor-derived EVs and exosomes have been so far identified. Their relevance in PC progression will be discussed in the subsequent sections of this review.

## EVs in prostate tissue and PC

### Prostasomes in prostate and PC

Prostate-derived EVs were found many years ago in prostatic fluids as well as seminal plasma, and hence named ‘prostasomes’ [24–26]. Although sharing many features with exosomes, such as the storage into (inside storage) with the consequent release from MVBs and the expression of exosome markers, such as CD9 and CD63, prostasomes appear larger (50–500 nm) than exosomes and may possess a multilayer membrane. Additionally, their lipidic content is mainly represented by sphingomyelin and their membranes contain high cholesterol levels [27]. Prostasomes might also contain chromosomal DNA, representing an array of small DNA fragments randomly selected from the entire human genome [28, 29], or derived from contaminating apoptotic bodies containing small DNA fragments [30].

In physiological conditions, prostasomes interact with spermatozoa and regulate sperm cell functions, such as spermatozoa motility, capacitation events, acrosome reaction, recognition by the female’s immune cells. They also exhibit antioxidant and antibacterial capacities. Therefore, the role of prostasomes in male fertility and reproduction is undeniable [30].

Hyperplastic prostate cells, PC and metastatic PC cells release prostasomes, which exhibit some typical properties of prostasomes derived from normal cells [31, 32]. However, once released from the apical side of the normal, columnar prostate epithelial cells into the glandular lumen, prostasomes can be detected in the semen or urine. By contrast, PC cells release prostasomes in the interstitial compartment or even into the bloodstream, since transformed cells lose their polarity and often invade the basement membrane [33, 34].

Many findings have pointed to the differences in marker’s expression or even to the different functions exhibited by prostasomes derived from normal or PC cells. Some of these characteristics include the involvement of PC-derived prostasomes in the complement pathway, the expression of surface enzymes and the promotion of angiogenesis that might influence PC progression [34]. In contrast, prostasome derived from the seminal fluid of healthy men inhibit angiogenesis [35, 36]. Additionally, prostasomes express the complement regulatory proteins, CD46 and CD59 and might transfer CD59 to the surrounding cells [37]. PC-derived prostasomes acquire the ability to transfer CD59, as compared to prostasomes from normal cells [38]. Because of the high kinase activity levels, PC-derived prostasomes phosphorylate the complement protein C3 and fibrinogen [39]. These changes are implicated in cancer cell motility [40]. Additionally, PC-derived prostasomes exhibit hyper-activation of enzymes involved in ECM degradation and cancer cell invasion, such as matrix metalloproteinases (MMP), plasminogen activator and peptidoglycan hydrolyzing enzymes [41]. At last, PC-derived prostasomes exhibit reduced levels of CD26, thus lowering its onco-suppressor function [41]. Of note, the TMPRSS2 serine protease is also secreted as component of the prostasomes into human semen [42]. The recent finding that TMPRSS2 activity is required for fusion between SARS-CoV-2 and human sperm has increased the interest in TMPRSS2 functions and paved the way for the sexual transmission of the virus [43]. Nonetheless, the role of TMPRSS2 in PC-derived prostasome still remains unclear, while its de-regulation by gene fusion is implicated in PC metastasis [44]. From the presented findings, it appears that prostasomes from PC cells foster survival and tumor progression. Their use in diagnostic approach of this disease is, hence, envisaged.

### Oncosomes in PC

Highly migratory PC cells release large EVs, (1–10 μm), which have been named “large oncosomes” (LO; 45). LO derive from the shedding of non-apoptotic plasma membrane blebs and their release is fostered by overexpression of oncoproteins, including the membrane-bound myristoylated-AKT, heparin-binding EGF-like growth factor (HB-EGF), caveolin-1 and epidermal growth factor receptor (EGF-R; 45–47) or the loss of cytoskeletal regulator diaphanous related formin-3 (DIAPH3), which controls the mesenchyme-amoeboid transition [48]. LO are released from amoeboid, highly invasive PC cells and can be detected in tumor specimens and plasma from patients or mice with metastatic PC. Notably, LO degrade ECM in vitro and cannot be detected in benign specimens [46]. By delivering signaling factors implicated in cell proliferation, growth and motility or RNA processing, they might activate specific molecular pathways. Additionally, LO are enriched of proteins and enzymes involved in glucose, glutamine as well as amino acid metabolism, and exhibit abundant levels of cytokeratin 18. These signatures suggest a role for LO in PC progression and identify them as a source of markers representative of PC malignancy [49]. It has been subsequently shown that once internalized by human normal fibroblasts, LO from patients with metastatic PC or PC cells induce the fibroblast reprogramming, enabling the endothelial tube formation in vitro and tumor growth in vivo*.* Dissection of molecular mechanism has revealed that LO from patients with metastatic PC or PC cells contain abundant levels of active AKT1, which is required for c-MYC activation in the surrounding stroma. From these data, it might be concluded that activation of stromal c-MYC is critical for re-programming of stromal cells, and that inhibition of LO internalization might impair the tumor-supporting properties of fibroblasts by preventing c-MYC activation [50]. In addition to providing evidence that LO mediate intercellular communication in aggressive PC, such mechanism offers new hints for future therapeutic application. More recently, it has been also reported that LO derived from invasive PC-derived cells express high levels of αV-integrin, which upon internalization by autocrine and/or paracrine loop, sustain the aggressive phenotype of PC cells, [51].

Taken together, the arguments put forward here add new insights into biological functions of LO in PC development and progression. Overall, they suggest that LO activity can be further exploited for identification of novel diagnostic markers and therapeutic targets in aggressive and metastatic PC.

### Exosomes in PC

Exosomes have emerged as a key communication mechanism between different cell types in the tumor microenvironment. They deliver important information from one cell to another and re-program the recipient cells, thus regulating their proliferation, survival and immune-surveillance. Most of the current studies have been obtained using exosomes derived from PC cells, or body fluids (plasma, serum, urine) from PC patients. The findings obtained from differential analyses are included in specific databases, such as Vesiclepedia (http://www.microvesiscles.org; 52) or ExoCarta (http://www.exocarta.org; 53), which represent important web-based compendia for exosomal research and content. The obtained information shows how exosomes are heterogeneous in PC and proteins as well as nucleic acids (miRNAs, mRNA, lncRNAs) can change depending on the PC stage.

In plasma of PC patients there is a higher number of exosomes than in healthy men or in patients with benign prostatic hyperplasia (BPH; 54, 55). Furthermore, the number of urinary exosomes, higher in PC patients, decreases after androgen deprivation therapy [56]. Besides the exosome’s enumeration, their molecular structure changes with the prostate status (BPH, PC or castration resistant PC). The lipidic composition is quite different, as exosomes from PC patients have a higher content in lactosylceramide and phosphatidylserine than vesicles from healthy controls [57].

The size and the tetraspanin content, in particular CD9, CD81, CD63 and CD41a are different in exosomes collected from benign prostatic hyperplasia, localized PC (LPC) and advanced prostate cancer (AdvPC; 58). BPH and AdvPC display a similar profile: CD63^+^ and CD81^+^ vesicles with a diameter of 50 nm, CD9^+^ with a diameter of both 50 and 55 nm and CD41a vesicles with a diameter of 60 nm. In LPC, CD63^+^ vesicles have mainly a diameter of 50 or 55 nm, while CD9^+^ has a diameter of 55 nm. The CD9^+^ vesicles are mostly represented in AdvPC than in BPH and LPC. Bigger differences aree highlighted when another marker, the prostate cancer specific PMSA is used to select exosomes. In this case, PSMA and CD9 positive vesicles are most abundant in AdvPC than in BPH or LPC while PSMA, CD9 and CD63 positive vesicles are most copious in AdvPC and LPc than in BPH. Again, vesicles CD9 and CD63 positive were highly represented in LPC than in BPH or LPC [58].

Concentration and type of nucleic acids and proteins loaded in exosomes change with the prostate health condition. MiR-375 and miR-141 expression is associated with the pathological stage and Gleason score [59] and their level is high in patients with castration resistant PC [60]. MiR-141 level is also used to distinguish patients with PC from healthy controls [61].

Nilsson and Colleagues demonstrated that exosome RNA content can be used as a diagnostic marker. In their study, all PC patients express PCA3, whereas only patients with high Gleason score and high PSA levels exhibit the mRNA transcript for the fusion gene TMPRSS2:ERG. In prostatectomized or ADT-treated patients with bone metastases, TMPRSS2:ERG and PCA3 were not detactable [62].

Some PC markers, such as PSA, PSMA and tumor-associated marker T54 have been detected only in PC urinary exosomes [56]. In PC patients, exosomes contain a higher level of survivin (a protein inhibiting apoptosis) than in patients with BPH or in healthy men. However, almost all cell types, from benign to malignant prostate tissue, release exosomes [63]. Since prostate cancer-surrounding stroma also releases exosomes [64] might be argued that exosomes from cancer cells and microenvironment cross-cooperate to promote PC malignancy.

Data from in vitro studies better analyze the role of proteins and miRNA carried by PC vesicles.

Exosomes from aggressive PC cells, such as PC3 and DU-145 cells, contain high levels of transforming growth factor-β (TGF-β). Once internalized by stromal cells, it induces activation of fibroblasts into myo-fibroblasts through the TGF-β/ mothers against decapentaplegic homolog 3 (SMAD3) signaling activation [65]. Again, exosomes from PC-derived cells or xenografted LNCaP cells or serum from PC patients contain high levels of EGFR, suggesting that detection of the receptor in exosomes from PC patients can be used as a marker of the disease state [66]. Using DU145 cells expressing a constitutively active EGFR, Read and Colleagues have reported that this EGFR mutant is trafficked through extracellular vesicles and transported to the nucleus of un-transfected DU145 [67]. These results have suggested that EGFR or its mutant versions can be transferred through exosomes, thereby modulating PC growth and progression. The finding that EGFR might be used as a marker for PC state is, however, arguable. Although data in cultured cells and xenografts, including ours, have indicated that EGFR plays a role in PC progression [68, 69], clinical trials with EGFR inhibitors have shown limited efficacy in PC patients [70, 71]. Again, in addition to expressing the full length 170 kDa EGFR, serum-derived exosomes from PC patients also express a variant protein migrating at 110 kDa, which likely represents a soluble form of EGFR. Nevertheless, the EGFR isoform detectable in PC patient serum does not exhibit to date a role in prostate tumorigenesis [66]. As such, further investigations are required for a better evaluation of EGFR and its isoforms in PC-derived exosomes. A recent study reported that exosomes from PC-derived 22Rv1 cells contain high levels of the hyaluronidase Hyal1, which is implicated in PC progression and metastasis. Exosome-derived Hyal1 stimulates, indeed, the migration of prostate stromal cells, simultaneously with an increase in adhesion to a type IV collagen matrix as well as focal adhesion kinase (FAK) phosphorylation and integrin engagement [72]. The presence of Hyal1 in PC-derived exosomes, together with its ability to impact the behavior of stromal cells, suggests that elevated Hyal1 levels in exosomes promote PC spreading and progression. Again, PC-derived exosomes are enriched of active Src and FAK as well as insulin-like growth factor I receptor (IGFI-R; 73). These findings provide new hints in biomarker detection by liquid biopsy. Of note, they indicate that PC-derived exosomes are enriched of signaling effectors commonly engaged by the androgen receptor (AR) to transmit its non-genomic effects in PC and stromal cells as well as cancer-associated fibroblasts (CAFs) from PC patients [11, 68, 74–77]. These considerations raise the question whether or not PC-derived exosomes contain AR, which still represents one of the most important targeted biomarkers in PC.

Some years ago, it was reported that exosomes secreted from LNCaP, but not PC3 cells contain AR. The Authors, however, failed to detect AR in exosomes derived from the plasma of PC patients, likely because of the low amounts of exosomes isolated from blood as well as the low content of AR in exosomes. However, the two markers, CD9 and CD63 were expressed in exosomes from LNCaP cells [54]. Subsequent results showed that CD9-enriched exosomes from PC cells or patients modulate the growth of androgen-deprived PC cells by paracrine signaling [78], indicating that PC-derived exosomes might influence the AR activity. Furthermore, the AR splice variant 7 (AR-V7) mRNA was isolated from exosome-RNA in the blood of PC patients, and it was associated with the resistance to the anti-androgens, enzalutamide or abiraterone [79]. More recently, AR amplification and AR-V7 mutant have been identified in circulating DNA or RNA of plasma-derived exosomes from PC patients [80]. Despite the small patient’s cohort analyzed in this study, the resulting findings would help to stratify PC patients who benefit from abiraterone or enzalutamide therapy, since AR amplification [81] and AR-V7 mutant [82] represent valuable biomarkers for treatment guidance in castration resistant PC (CRPC). Additionally, three AR aberrations (AR T878A, AR-V7 and wild-type AR amplification) have been recently identified in enriched-tumor exosomes from a small cohort of CRPC patients. Here again, the exosome cargo has been linked to response or resistance to treatment with anti-androgens [83]. A very recent study reinforced the concept that exosome-RNA represents a reliable source of AR variants, which might predict the PC-resistance to AR signaling inhibitors [84]. In summary, these studies have explored the advantage of a feasible approach to stratify CRPC patients in the context of their response to anti-androgen therapy. Nevertheless, they have left still pending the question concerning the impact of exosome-derived AR (full-length or mutants) in PC progression and metastasis. By using exosomes derived from 22Rv1 cells, which contain both the full-lenght AR as well as the AR-V7 mutant, it has been shown that exosome cargo can be directly transferred to the nuclear compartment of AR-negative PC3 cells, thus activating gene transcription [67]. Therefore, further investigations of the mechanisms regulating the receptor transfer through exosomes might offer unexpected advances in the understanding of PC progression. However, a precise  picture of the role of AR or its variants in exosomes still appears tricky, likely because the approaches so far used (cell culture in vitro, xenograft experiments in mouse, correlative studies of exosome-derived AR with therapeutic response in PC patients) have hindered the progress in this topic.

The past years have seen a great expansion into CAFs researches. These cells release ECM, growth factors, cytokines and exosomes that might promote tumor progression. CAFs-derived exosomes can transfer proteins, messenger RNAs (mRNAs), and microRNAs (miRNAs). Their putative effects in PC have been extensively discussed [85] and several studies have investigated the miRNA profile in serum/plasma from PC patients [59, 61, 86, 87].

The role of CAFs in metabolic reprogramming of PC cells is largely recognized. Once activated, CAFs from PC patients produce lactate, which shuttles back to PC cells, fueling their proliferation [88]. Subsequent studies from the same group have shown that CAFs directly transfer their functional mitochondria to PC cells, further promoting mitochondrial utilization by cancer cells and PC malignancy [89]. These studies have revealed intriguing and novel aspects of CAFs secretoma. The role of exosomes in metabolic reprogramming of PC cells has been investigated in CAFs exosomes derived from PC patients, and it has been shown that they provide nutrients that enable PC growth, even under nutrient deprivation or nutrient stressed conditions [90]. Altogether, these findings pave the way for new therapeutic approaches targeting the communication exchange between CAFs, CAF-derived exosomes and PC cells.

Beyond the well-established role of miRNA and exosomal miRNA in PC cells and tissues [85, 91], many findings have shown that transfer of miRNAs through EVs from the CAFs to the adjacent epithelial cells induces tumor growth in several PC mouse models [92, 93]. This transfer promotes EMT of PC cells, thereby increasing their migration, invasion, and metastasis to bone and soft tissues. Studies of miRNA members, miR-409, miR-379 and miR-154*, located within the delta-like 1 homolog-deiodinase, iodothyronine 3 (DLK1-DIO3) imprinted region located on human chromosome 14, have shown that these miRNAs induce tumor effects in vitro and in PC xenograft models. DLK1-DIO3 miRNAs are “hijacked” to promote PC tumorigenesis and metastasis through enhancement of tumor–stroma interactions. Since PC cells are susceptible to activation by surrounding cells, this process activates pathways that lead to enhanced growth, survival and metastasis of PC [92, 93]. Again, a recent study reported that CAFs exosomes from PC patients over-express miR-423-5p, which mediates resistance of PC cells to taxanes [94]. Taken together, the findings here discussed indicate that DLK1-DIO3 miRNAs are attractive therapeutic targets to block the tumor–stroma interactions in PC patients and demonstrate a role for miR-423-5p in PC chemoresistance.

**Fig. 2:**
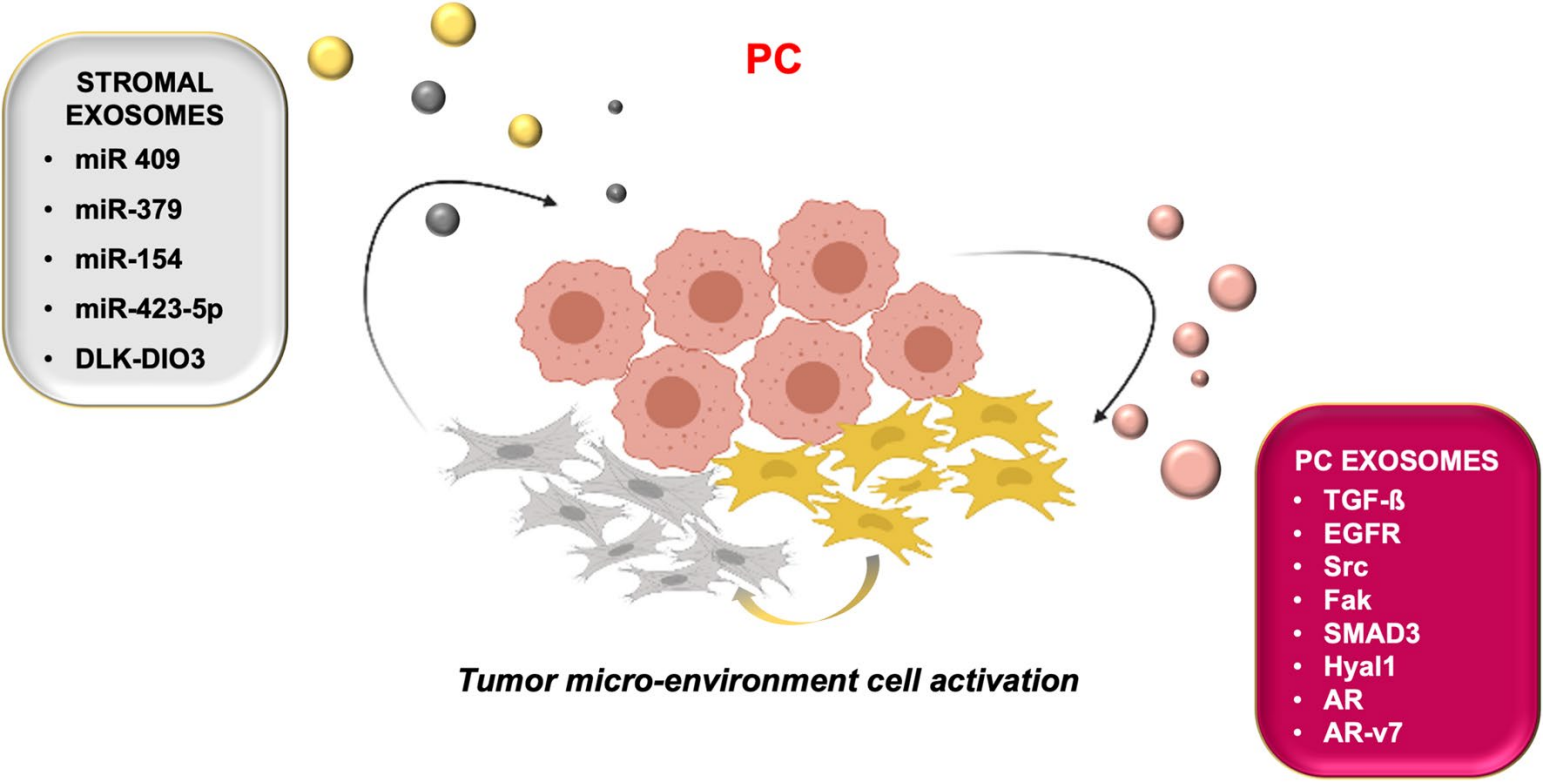
The figure summarizes the content of stromal and prostate cancer exosomes. The released cargoes might be responsible for PC progression and drug-resistance

In conclusion, PC arises and progresses through an intricate network of signals and information exchanged by cancerous cells and the surrounding microenvironment. The complexity of this network relies on the ability of EVs to transfer, both locally and systemically, a repertoire of bioactive molecules regulating PC malignancy and drug-resistance (Fig. 2). Therefore, exosomes represent promising circulating biomarkers detectable through non-invasive liquid biopsy for early diagnosis, screening and risk of PC relapses. Their shuttled signaling might be specifically targeted to interrupt cell-to-cell communication in PC.

### Androgens, anti-androgens and exosomes

In literature, only few studies have analyzed the effect of androgens and anti-androgens on the heterogeneity of exosomes in PC.

Extracellular vesicles positive for CD9 or double positive for CD9 and PMSA are the most expressed in patients with advanced metastatic PC, while there is a higher number of exosomes double positive for CD9 and CD63 in patients with localised PC [58]. Dihydrotestesterone (DHT) and Enzalutamide alter size and heterogeneity of CD9 exosomes in AR positive PC cells. The androgen and its antagonists have no effect on number and type of protein cargo, but influence the RNA content of vesicles. Five hundred and forty-three small RNA are controlled by androgen treatment including miR-19-3p and miR-361-5p [58].

In another study, McCormick and Colleagues demonstrated that the anti-androgen Casodex stimulates the secretion of exosomes characterized by the expression of Rab11a in CRPC in vitro models. In these cells, the antiandrogen reduces the mTORC1 activity, thus promoting the Rab11a-exosome release [95]. Similar data were obtained in aromatase inhibitor resistant breast cancer cells, in which the upregulated exosome production is due to an enhanced RabGTPase expression [96]. It is known that other GTPases, such as Rac, Rho and Ras are controlled by androgens [77, 97] and are involved in exosomes biogenesis [98, 99]. Thus, it could be intriguing to scrutinize this aspect in the future.

### Clinical and therapeutic use of exosomes

Different studies highlight the pivotal role of exosomes in PC development and progression. By carrying different molecules, exosomes allow PC cells to communicate with tumor microenvironment, thus creating the best niche both for primary and metastatic cancer. Furthermore, considering that exosome's composition in protein and nucleic acids depends on cancer stage or cancer response to chemical treatment, these vesicles represent a potential source of tumor biomarkers for both diagnosing the PC stages and monitoring PC therapies. Exosomes are detectable in blood, urine, prostatic secretions, saliva and other biological fluids, for this they can be quickly collected with a minimal burden for the patients, contrary to the prostatic biopsies now used.

Nevertheless, cause of their reduced shape and their ability to fuse with cancer cells, biocompatibility, stability, low immunogenicity and tossicity, exosomes can be engineered to deliver proteins, short interfering RNA, long non-coding RNA, microRNA, nucleic acids, immune modulators, and drugs to therapeutic purpose [15]. As proposed by Batracova et al. [100], exosomes represent a promising tool in cancer chemotherapy. Thanks to their structure, exosomes can penetrate organs protected by physiological barriers and can circumvent the immune system of cells without being destroyed and are also well tolerated [101].

Obviously, their use as therapeutic target needs to be better investigated, but represents a promising challenge.

## Concluding remarks

PC remains a major public health problem. Given the expected increase in PC burden, it is a priority to rapidly translate basic research findings into novel and effective tools for the early detection, diagnosis and prognosis in PC patients.

Many advances have been made in this direction. Nevertheless, pieces of the PC puzzle are still missing, especially concerning the complexities of cell-to-cell communication. Preclinical and clinical findings here discussed might provide new insights into the molecular characteristics of PC- and stromal-derived exosomes. They may also have many potentialities for future applications in PC therapy as well as other common endocrine-related malignancies, such as breast and thyroid cancers.

## Data Availability

Not applicable.

## Abbreviations

AR: Androgen receptor
CAFs: Cancer-associated fibroblasts
CRPC: Castrate resistant prostate cancer
CTCs: Circulating tumor cells
DIAPH3: Diaphanous related formin-3
DLK1-DIO3: Located within the delta-like 1 homolog-deiodinase, iodothyronine 3
ECM: Extracellular matrix
EGF-R: Epidermal growth factor receptor
EMT: Epithelial-mesenchyme transition
ESCRT: Endosomal sorting complexes required for transport
EVs: Extracellular vesicles
FAK: Focal adhesion kinase
HB-EGF: Heparin-binding EGF-like growth factor
IGFI-R: Insulin-like growth factor I receptor
ILVs: Intraluminal vesicles
LO: Large oncosomes
MICs: Metastases-initiating cells
MVBs: Intracellular multivesicular bodies
MMP: Matrix metalloproteinase
PC: Prostate cancer
SARS-CoV-2: Severe acute respiratory syndrome coronavirus 2
SMAD3: Mothers against decapentaplegic homolog 3
TGF-β: Transforming growth factor-β
TMPRSS2: Transmembrane protease, serine 2

## References

[1] Di Zazzo E, Galasso G, Giovannelli P, Di Donato M, Castoria G (2018). Estrogens and their receptors in prostate cancer: therapeutic implications. Front Oncol.

[2] Di Zazzo E, Galasso G, Giovannelli P, Di Donato M, Bilancio A, Perillo B, Sinisi AA, Migliaccio A, Castoria G (2019). Estrogen receptors in epithelial-mesenchymal transition of prostate cancer. Cancers (Basel).

[3] Shiao SL, Chu GC, Chung LW (2016). Regulation of prostate cancer progression by the tumor microenvironment. Cancer Lett.

[4] Olumi AF, Grossfeld GD, Hayward SW, Carroll PR, Tlsty TD, Cunha GR (1999). Carcinoma-associated fibroblasts direct tumor progression of initiated human prostatic epithelium. Cancer Res.

[5] Delsite R, Djakiew D (1999). Characterization of nerve growth factor precursor protein expression by human prostate stromal cells: a role in selective neurotrophin stimulation of prostate epithelial cell growth. Prostate.

[6] Bhowmick NA, Neilson EG, Moses HL (2004). Stromal fibroblasts in cancer initiation and progression. Nature.

[7] Green SM, Mostaghel EA, Nelson PS (2012). Androgen action and metabolism in prostate cancer. Mol Cell Endocrinol.

[8] Bruzzese F, Hägglöf C, Leone A, Sjöberg E, Roca MS, Kiflemariam S, Sjöblom T, Hammarsten P, Egevad L, Bergh A, Ostman A, Budillon A, Augsten M (2014). Local and systemic protumorigenic effects of cancer-associated fibroblast-derived GDF15. Cancer Res.

[9] Cioni B, Nevedomskaya E, Melis MHM, van Burgsteden J, Stelloo S, Hodel E, Spinozzi D, de Jong J, van der Poel H, de Boer JP, Wessels LFA, Zwart W, Bergman AM (2018). Loss of androgen receptor signaling in prostate cancer-associated fibroblasts (CAFs) promotes CCL2- and CXCL8-mediated cancer cell migration. Mol Oncol.

[10] Parri M, Ippolito L, Cirri P, Ramazzotti M, Chiarugi P (2020). Metabolic cell communication within tumour microenvironment: models, methods and perspectives. Curr Opin Biotechnol.

[11] Di Donato M, Zamagni A, Galasso G, Di Zazzo E, Giovannelli P, Barone MV, Zanoni M, Gunelli R, Costantini M, Auricchio F, Migliaccio A, Tesei A, Castoria G (2021). The androgen receptor/filamin A complex as a target in prostate cancer microenvironment. Cell Death Dis.

[12] Tkach M, Théry C (2016). Communication by extracellular vesicles: where we are and where we need to go. Cell.

[13] Webber J, Yeung V, Clayton A (2015). Extracellular vesicles as modulators of the cancer microenvironment. Semin Cell Dev Biol.

[14] Becker A, Thakur BK, Weiss JM, Kim HS, Peinado H, Lyden D (2016). Extracellular vesicles in cancer: cell-to-cell mediators of metastasis. Cancer Cell.

[15] Kalluri R, LeBleu VS (2020). The biology, function, and biomedical applications of exosomes. Science.

[16] Cocucci E, Meldolesi J (2015). Ectosomes and exosomes: shedding the confusion between extracellular vesicles. Trends Cell Biol.

[17] Tkach M, Kowal J, Théry C (2018). Why the need and how to approach the functional diversity of extracellular vesicles. Philos Trans R Soc Lond B Biol Sci.

[18] Pegtel DM, Gould SJ (2019). Exosomes. Annu Rev Biochem.

[19] Baj-Krzyworzeka M, Szatanek R, Weglarczyk K, Baran J, Urbanowicz B, Brański P, Ratajczak MZ, Zembala M (2006). Tumour-derived microvesicles carry several surface determinants and mRNA of tumour cells and transfer some of these determinants to monocytes. Cancer Immunol Immunother.

[20] Ratajczak J, Wysoczynski M, Hayek F, Janowska-Wieczorek A, Ratajczak MZ (2006). Membrane-derived microvesicles: important and underappreciated mediators of cell-to-cell communication. Leukemia.

[21] Valadi H, Ekström K, Bossios A, Sjöstrand M, Lee JJ, Lötvall JO (2007). Exosome-mediated transfer of mRNAs and microRNAs is a novel mechanism of genetic exchange between cells. Nat Cell Biol.

[22] Kim DK, Kang B, Kim OY, Choi DS, Lee J, Kim SR, Go G, Yoon YJ, Kim JH, Jang SC, Park KS, Choi EJ, Kim KP, Desiderio DM, Kim YK, Lötvall J, Hwang D, Gho YS (2013). EVpedia: an integrated database of high-throughput data for systemic analyses of extracellular vesicles. J Extracell Vesicles.

[23] Al-Nedawi K, Meehan B, Micallef J, Lhotak V, May L, Guha A, Rak J (2008). Intercellular transfer of the oncogenic receptor EGFRvIII by microvesicles derived from tumour cells. Nat Cell Biol.

[24] Ronquist G, Hedström M (1977). Restoration of detergent-inactivated adenosine triphosphatase activity of human prostatic fluid with concanavalin A. Biochim Biophys Acta.

[25] Ronquist G, Brody I, Gottfries A, Stegmayr B (1978). An Mg2+ and Ca2+-stimulated adenosine triphosphatase in human prostatic fluid: part I. Andrologia.

[26] Ronquist G, Brody I, Gottfries A, Stegmayr B (1978). An Mg^2+^ and Ca^2+^-stimulated adenosine triphosphatase in human prostatic fluid–part II. Andrologia.

[27] Arienti G, Carlini E, De Cosmo AM, Di Profio P, Palmerini CA (1998). Prostasome-like particles in stallion semen. Biol Reprod.

[28] Ronquist KG, Ronquist G, Carlsson L, Larsson A (2009). Human prostasomes contain chromosomal DNA. Prostate.

[29] Ronquist GK, Larsson A, Ronquist G, Isaksson A, Hreinsson J, Carlsson L, Stavreus-Evers A (2011). Prostasomal DNA characterization and transfer into human sperm. Mol Reprod Dev.

[30] Aalberts M, Stout TA, Stoorvogel W (2013). Prostasomes: extracellular vesicles from the prostate. Reproduction.

[31] Carlsson L, Nilsson O, Larsson A, Stridsberg M, Sahlén G, Ronquist G (2003). Characteristics of human prostasomes isolated from three different sources. Prostate.

[32] Sahlén G, Ahlander A, Frost A, Ronquist G, Norlén BJ, Nilsson BO (2004). Prostasomes are secreted from poorly differentiated cells of prostate cancer metastases. Prostate.

[33] Sahlén GE, Egevad L, Ahlander A, Norlén BJ, Ronquist G, Nilsson BO (2002). Ultrastructure of the secretion of prostasomes from benign and malignant epithelial cells in the prostate. Prostate.

[34] Ronquist G, Nilsson BO (2004). The Janus-faced nature of prostasomes: their pluripotency favours the normal reproductive process and malignant prostate growth. Prostate Cancer Prostatic Dis.

[35] Delves GH, Stewart AB, Lwaleed BA, Cooper AJ (2005). In vitro inhibition of angiogenesis by prostasomes. Prostate Cancer Prostatic Dis.

[36] Delves GH, Goyal A, Lwaleed BA, Cooper AJ (2006). Seminal prostasomes inhibit the angiogenesis activity of rat aortic rings. Prostate Cancer Prostatic Dis.

[37] Kitamura M, Namiki M, Matsumiya K, Tanaka K, Matsumoto M, Hara T, Kiyohara H, Okabe M, Okuyama A, Seya T (1995). Membrane cofactor protein (CD46) in seminal plasma is a prostasome-bound form with complement regulatory activity and measles virus neutralizing activity. Immunology.

[38] Babiker AA, Nilsson B, Ronquist G, Carlsson L, Ekdahl KN (2005). Transfer of functional prostasomal CD59 of metastatic prostatic cancer cell origin protects cells against complement attack. Prostate.

[39] Babiker AA, Ronquist G, Nilsson B, Ekdahl KN (2006). Overexpression of ecto-protein kinases in prostasomes of metastatic cell origin. Prostate.

[40] Simpson-Haidaris PJ, Rybarczyk B (2001). Tumors and fibrinogen. The role of fibrinogen as an extracellular matrix protein. Ann N Y Acad Sci.

[41] Bellezza I, Aisa MC, Palazzo R, Costanzi E, Mearini E, Minelli A (2005). Extracellular matrix degrading enzymes at the prostasome surface. Prostate Cancer Prostatic Dis.

[42] Chen YW, Lee MS, Lucht A, Chou FP, Huang W, Havighurst TC, Kim K, Wang JK, Antalis TM, Johnson MD, Lin CY (2010). TMPRSS2, a serine protease expressed in the prostate on the apical surface of luminal epithelial cells and released into semen in prostasomes, is misregulated in prostate cancer cells. Am J Pathol.

[43] He W, Liu X, Feng L, Xiong S, Li Y, Chen L, Li Y, Wang G, Li D, Fu B (2020). Impact of SARS-CoV-2 on male reproductive health: a review of the literature on male reproductive involvement in COVID-19. Front Med (Lausanne).

[44] Rebello RJ, Oing C, Knudsen KE, Loeb S, Johnson DC, Reiter RE, Gillessen S, Van der Kwast T, Bristow RG (2021). Prostate cancer. Nat Rev Dis Primers.

[45] Di Vizio D, Kim J, Hager MH, Morello M, Yang W, Lafargue CJ, True LD, Rubin MA, Adam RM, Beroukhim R, Demichelis F, Freeman MR (2009). Oncosome formation in prostate cancer: association with a region of frequent chromosomal deletion in metastatic disease. Cancer Res.

[46] Di Vizio D, Morello M, Dudley AC, Schow PW, Adam RM, Morley S, Mulholland D, Rotinen M, Hager MH, Insabato L, Moses MA, Demichelis F, Lisanti MP, Wu H, Klagsbrun M, Bhowmick NA, Rubin MA, D'Souza-Schorey C, Freeman MR (2012). Large oncosomes in human prostate cancer tissues and in the circulation of mice with metastatic disease. Am J Pathol.

[47] Kim J, Morley S, Le M, Bedoret D, Umetsu DT, Di Vizio D, Freeman MR (2014). Enhanced shedding of extracellular vesicles from amoeboid prostate cancer cells: potential effects on the tumor microenvironment. Cancer Biol Ther.

[48] Hager MH, Morley S, Bielenberg DR, Gao S, Morello M, Holcomb IN, Liu W, Mouneimne G, Demichelis F, Kim J, Solomon KR, Adam RM, Isaacs WB, Higgs HN, Vessella RL, Di Vizio D, Freeman MR (2012). DIAPH3 governs the cellular transition to the amoeboid tumour phenotype. EMBO Mol Med.

[49] Minciacchi VR, You S, Spinelli C, Morley S, Zandian M, Aspuria PJ, Cavallini L, Ciardiello C, Reis Sobreiro M, Morello M, Kharmate G, Jang SC, Kim DK, Hosseini-Beheshti E, Tomlinson Guns E, Gleave M, Gho YS, Mathivanan S, Yang W, Freeman MR, Di Vizio D (2015). Large oncosomes contain distinct protein cargo and represent a separate functional class of tumor-derived extracellular vesicles. Oncotarget.

[50] Minciacchi VR, Spinelli C, Reis-Sobreiro M, Cavallini L, You S, Zandian M, Li X, Mishra R, Chiarugi P, Adam RM, Posadas EM, Viglietto G, Freeman MR, Cocucci E, Bhowmick NA, Di Vizio D (2017). MYC mediates large oncosome-induced fibroblast reprogramming in prostate cancer. Cancer Res.

[51] Ciardiello C, Leone A, Lanuti P, Roca MS, Moccia T, Minciacchi VR, Minopoli M, Gigantino V, De Cecio R, Rippa M, Petti L, Capone F, Vitagliano C, Milone MR, Pucci B, Lombardi R, Iannelli F, Di Gennaro E, Bruzzese F, Marchisio M, Carriero MV, Di Vizio D, Budillon A (2019). Large oncosomes overexpressing integrin alpha-V promote prostate cancer adhesion and invasion via AKT activation. J Exp Clin Cancer Res.

[52] Kalra H, Simpson RJ, Ji H, Aikawa E, Altevogt P, Askenase P, Bond VC, Borràs FE, Breakefield X, Budnik V, Buzas E, Camussi G, Clayton A, Cocucci E, Falcon-Perez JM, Gabrielsson S, Gho YS, Gupta D, Harsha HC, Hendrix A, Hill AF, Inal JM, Jenster G, Krämer-Albers EM, Lim SK, Llorente A, Lötvall J, Marcilla A, Mincheva-Nilsson L, Nazarenko I, Nieuwland R, Nolte-'t Hoen EN, Pandey A, Patel T, Piper MG, Pluchino S, Prasad TS, Rajendran L, Raposo G, Record M, Reid GE, Sánchez-Madrid F, Schiffelers RM, Siljander P, Stensballe A, Stoorvogel W, Taylor D, Thery C, Valadi H, van Balkom BW, Vázquez J, Vidal M, Wauben MH, Yáñez-Mó M, Zoeller M, Mathivanan S (2012). Vesiclepedia: a compendium for extracellular vesicles with continuous community annotation. PLoS Biol.

[53] Keerthikumar S, Chisanga D, Ariyaratne D, Al Saffar H, Anand S, Zhao K, Samuel M, Pathan M, Jois M, Chilamkurti N, Gangoda L, Mathivanan S (2016). ExoCarta: a web-based compendium of exosomal cargo. J Mol Biol.

[54] Mizutani K, Terazawa R, Kameyama K, Kato T, Horie K, Tsuchiya T, Seike K, Ehara H, Fujita Y, Kawakami K, Ito M, Deguchi T (2014). Isolation of prostate cancer-related exosomes. Anticancer Res.

[55] Turay D, Khan S, Diaz Osterman CJ, Curtis MP, Khaira B, Neidigh JW, Mirshahidi S, Casiano CA, Wall NR (2016). Proteomic profiling of serum-derived exosomes from ethnically diverse prostate cancer patients. Cancer Investig.

[56] Mitchell PJ, Welton J, Staffurth J, Court J, Mason MD, Tabi Z, Clayton A (2009). Can urinary exosomes act as treatment response markers in prostate cancer?. J Transl Med.

[57] Skotland T, Ekroos K, Kauhanen D, Simolin H, Seierstad T, Berge V, Sandvig K, Llorente A (2017). Molecular lipid species in urinary exosomes as potential prostate cancer biomarkers. Eur J Cancer.

[58] Martens-Uzunova ES, Kusuma GD, Crucitta S, Lim HK, Cooper C, Riches JE, Azad A, Ochiya T, Boyle GM, Southey MC, Del Re M, Lim R, Ramm GA, Jenster GW, Soekmadji C (2021). Androgens alter the heterogeneity of small extracellular vesicles and the small RNA cargo in prostate cancer. J Extracell Vesicles.

[59] Brase JC, Johannes M, Schlomm T, Fälth M, Haese A, Steuber T, Beissbarth T, Kuner R, Sültmann H (2011). Circulating miRNAs are correlated with tumor progression in prostate cancer. Int J Cancer.

[60] Nguyen HC, Xie W, Yang M, Hsieh CL, Drouin S, Lee GS, Kantoff PW (2013). Expression differences of circulating microRNAs in metastatic castration resistant prostate cancer and low-risk, localized prostate cancer. Prostate.

[61] Mitchell PS, Parkin RK, Kroh EM, Fritz BR, Wyman SK, Pogosova-Agadjanyan EL, Peterson A, Noteboom J, O'Briant KC, Allen A, Lin DW, Urban N, Drescher CW, Knudsen BS, Stirewalt DL, Gentleman R, Vessella RL, Nelson PS, Martin DB, Tewari M (2008). Circulating microRNAs as stable blood-based markers for cancer detection. Proc Natl Acad Sci U S A.

[62] Nilsson J, Skog J, Nordstrand A, Baranov V, Mincheva-Nilsson L, Breakefield XO, Widmark A (2009). Prostate cancer-derived urine exosomes: a novel approach to biomarkers for prostate cancer. Br J Cancer.

[63] Vlaeminck-Guillem V (2018). Extracellular vesicles in prostate cancer carcinogenesis, diagnosis, and management. Front Oncol.

[64] Colombo M, Raposo G, Théry C (2014). Biogenesis, secretion, and intercellular interactions of exosomes and other extracellular vesicles. Annu Rev Cell Dev Biol.

[65] Webber J, Steadman R, Mason MD, Tabi Z, Clayton A (2010). Cancer exosomes trigger fibroblast to myofibroblast differentiation. Cancer Res.

[66] Kharmate G, Hosseini-Beheshti E, Caradec J, Chin MY, Tomlinson Guns ES (2016). Epidermal growth factor receptor in prostate cancer derived exosomes. PLoS ONE.

[67] Read J, Ingram A, Al Saleh HA, Platko K, Gabriel K, Kapoor A, Pinthus J, Majeed F, Qureshi T, Al-Nedawi K (2017). Nuclear transportation of exogenous epidermal growth factor receptor and androgen receptor via extracellular vesicles. Eur J Cancer.

[68] Migliaccio A, Varricchio L, De Falco A, Castoria G, Arra C, Yamaguchi H, Ciociola A, Lombardi M, Di Stasio R, Barbieri A, Baldi A, Barone MV, Appella E, Auricchio F (2007). Inhibition of the SH3 domain-mediated binding of Src to the androgen receptor and its effect on tumor growth. Oncogene.

[69] Migliaccio A, Castoria G, Di Domenico M, Ciociola A, Lombardi M, De Falco A, Nanayakkara M, Bottero D, De Stasio R, Varricchio L, Auricchio F (2006). Crosstalk between EGFR and extranuclear steroid receptors. Ann N Y Acad Sci.

[70] Wilding G, Soulie P, Trump D, Das-Gupta A, Small E (2006). Results from a pilot Phase I trial of gefitinib combined with docetaxel and estramustine in patients with hormone-refractory prostate cancer. Cancer.

[71] Sridhar SS, Hotte SJ, Chin JL, Hudes GR, Gregg R, Trachtenberg J, Wang L, Tran-Thanh D, Pham NA, Tsao MS, Hedley D, Dancey JE, Moore MJ (2010). A multicenter phase II clinical trial of lapatinib (GW572016) in hormonally untreated advanced prostate cancer. Am J Clin Oncol.

[72] McAtee CO, Booth C, Elowsky C, Zhao L, Payne J, Fangman T, Caplan S, Henry MD, Simpson MA (2019). Prostate tumor cell exosomes containing hyaluronidase Hyal1 stimulate prostate stromal cell motility by engagement of FAK-mediated integrin signaling. Matrix Biol.

[73] DeRita RM, Zerlanko B, Singh A, Lu H, Iozzo RV, Benovic JL, Languino LR (2017). c-Src, insulin-like growth factor I receptor, G-protein-coupled receptor kinases and focal adhesion kinase are enriched into prostate cancer cell exosomes. J Cell Biochem.

[74] Migliaccio A, Castoria G, Di Domenico M, de Falco A, Bilancio A, Lombardi M, Barone MV, Ametrano D, Zannini MS, Abbondanza C, Auricchio F (2000). Steroid-induced androgen receptor-oestradiol receptor beta-Src complex triggers prostate cancer cell proliferation. EMBO J.

[75] Migliaccio A, Di Domenico M, Castoria G, Nanayakkara M, Lombardi M, de Falco A, Bilancio A, Varricchio L, Ciociola A, Auricchio F (2005). Steroid receptor regulation of epidermal growth factor signaling through Src in breast and prostate cancer cells: steroid antagonist action. Cancer Res.

[76] Genua M, Pandini G, Sisci D, Castoria G, Maggiolini M, Vigneri R, Belfiore A (2009). Role of cyclic AMP response element-binding protein in insulin-like growth factor-i receptor up-regulation by sex steroids in prostate cancer cells. Cancer Res.

[77] Castoria G, D'Amato L, Ciociola A, Giovannelli P, Giraldi T, Sepe L, Paolella G, Barone MV, Migliaccio A, Auricchio F (2011). Androgen-induced cell migration: role of androgen receptor/filamin A association. PLoS ONE.

[78] Soekmadji C, Corcoran NM, Oleinikova I, Jovanovic L, Ramm GA, Nelson CC, Jenster G, Russell PJ, Australian Prostate Cancer Collaboration BioResource (2017). Extracellular vesicles for personalized therapy decision support in advanced metastatic cancers and its potential impact for prostate cancer. Prostate.

[79] Taneja SS (2015). Re: AR-V7 and resistance to enzalutamide and abiraterone in prostate cancer. J Urol.

[80] Del Re M, Crucitta S, Sbrana A, Rofi E, Paolieri F, Gianfilippo G, Galli L, Falcone A, Morganti R, Porta C, Efstathiou E, van Schaik R, Jenster G, Danesi R (2019). AR-V7 and AR-FL expression is associated with clinical outcome: a translational study in patients with castrate resistant prostate cancer. BJU Int.

[81] Romanel A, Gasi Tandefelt D, Conteduca V, Jayaram A, Casiraghi N, Wetterskog D, Salvi S, Amadori D, Zafeiriou Z, Rescigno P, Bianchini D, Gurioli G, Casadio V, Carreira S, Goodall J, Wingate A, Ferraldeschi R, Tunariu N, Flohr P, De Giorgi U, de Bono JS, Demichelis F, Attard G (2015). Plasma AR and abiraterone-resistant prostate cancer. Sci Transl Med.

[82] Antonarakis ES, Lu C, Luber B, Wang H, Chen Y, Zhu Y, Silberstein JL, Taylor MN, Maughan BL, Denmeade SR, Pienta KJ, Paller CJ, Carducci MA, Eisenberger MA, Luo J (2017). Clinical significance of androgen receptor splice variant-7 mRNA detection in circulating tumor cells of men with metastatic castration-resistant prostate cancer treated with first- and second-line abiraterone and enzalutamide. J Clin Oncol.

[83] Foroni C, Zarovni N, Bianciardi L, Bernardi S, Triggiani L, Zocco D, Venturella M, Chiesi A, Valcamonico F, Berruti A (2020). When less is more: specific capture and analysis of tumor exosomes in plasma increases the sensitivity of liquid biopsy for comprehensive detection of multiple androgen receptor phenotypes in advanced prostate cancer patients. Biomedicines.

[84] Del Re M, Conteduca V, Crucitta S, Gurioli G, Casadei C, Restante G, Schepisi G, Lolli C, Cucchiara F, Danesi R, De Giorgi U (2021). Androgen receptor gain in circulating free DNA and splicing variant 7 in exosomes predict clinical outcome in CRPC patients treated with abiraterone and enzalutamide. Prostate Cancer Prostatic Dis.

[85] Hessvik NP, Sandvig K, Llorente A (2013). Exosomal miRNAs as biomarkers for prostate cancer. Front Genet.

[86] Lodes MJ, Caraballo M, Suciu D, Munro S, Kumar A, Anderson B (2009). Detection of cancer with serum miRNAs on an oligonucleotide microarray. PLoS ONE.

[87] Moltzahn F, Olshen AB, Baehner L, Peek A, Fong L, Stöppler H, Simko J, Hilton JF, Carroll P, Blelloch R (2011). Microfluidic-based multiplex qRT-PCR identifies diagnostic and prognostic microRNA signatures in the sera of prostate cancer patients. Cancer Res.

[88] Fiaschi T, Marini A, Giannoni E, Taddei ML, Gandellini P, De Donatis A, Lanciotti M, Serni S, Cirri P, Chiarugi P (2012). Reciprocal metabolic reprogramming through lactate shuttle coordinately influences tumor-stroma interplay. Cancer Res.

[89] Ippolito L, Morandi A, Taddei ML, Parri M, Comito G, Iscaro A, Raspollini MR, Magherini F, Rapizzi E, Masquelier J, Muccioli GG, Sonveaux P, Chiarugi P, Giannoni E (2019). Cancer-associated fibroblasts promote prostate cancer malignancy via metabolic rewiring and mitochondrial transfer. Oncogene.

[90] Zhao H, Yang L, Baddour J, Achreja A, Bernard V, Moss T, Marini JC, Tudawe T, Seviour EG, San Lucas FA, Alvarez H, Gupta S, Maiti SN, Cooper L, Peehl D, Ram PT, Maitra A, Nagrath D (2016). Tumor microenvironment derived exosomes pleiotropically modulate cancer cell metabolism. Elife.

[91] Zhang W, Zang J, Jing X, Sun Z, Yan W, Yang D, Shen B, Guo F (2014). Identification of candidate miRNA biomarkers from miRNA regulatory network with application to prostate cancer. J Transl Med.

[92] Josson S, Gururajan M, Hu P, Shao C, Chu GY, Zhau HE, Liu C, Lao K, Lu CL, Lu YT, Lichterman J, Nandana S, Li Q, Rogatko A, Berel D, Posadas EM, Fazli L, Sareen D, Chung LW (2014). miR-409–3p/-5p promotes tumorigenesis, epithelial-to-mesenchymal transition, and bone metastasis of human prostate cancer. Clin Cancer Res.

[93] Josson S, Gururajan M, Sung SY, Hu P, Shao C, Zhau HE, Liu C, Lichterman J, Duan P, Li Q, Rogatko A, Posadas EM, Haga CL, Chung LW (2015). Stromal fibroblast-derived miR-409 promotes epithelial-to-mesenchymal transition and prostate tumorigenesis. Oncogene.

[94] Shan G, Gu J, Zhou D, Li L, Cheng W, Wang Y, Tang T, Wang X (2020). Cancer-associated fibroblast-secreted exosomal miR-423–5p promotes chemotherapy resistance in prostate cancer by targeting GREM2 through the TGF-β signaling pathway. Exp Mol Med.

[95] McCormick K, Sanitt P, Fan SH, Mason JD, Harris AL, Hamdy FC, Verrill C, Bryant RJ, Goberdhan DCI (2020). Anti-androgens induce Rab11a-exosome secretion in prostate cancer by suppressing amino acid-sensitive PAT4-mTORC1 signalling. BioRxiv.

[96] Augimeri G, La Camera G, Gelsomino L, Giordano C, Panza S, Sisci D, Morelli C, Győrffy B, Bonofiglio D, Andò S, Barone I, Catalano S (2020). Evidence for enhanced exosome production in aromatase inhibitor-resistant breast cancer cells. Int J Mol Sci.

[97] Castoria G, Giovannelli P, Di Donato M, Ciociola A, Hayashi R, Bernal F, Appella E, Auricchio F, Migliaccio A (2014). Role of non-genomic androgen signalling in suppressing proliferation of fibroblasts and fibrosarcoma cells. Cell Death Dis.

[98] Sexton RE, Mpilla G, Kim S, Philip PA, Azmi AS (2019). Ras and exosome signaling. Semin Cancer Biol.

[99] Ghoroghi S, Mary B, Larnicol A, Asokan N, Klein A, Osmani N, Busnelli I, Delalande F, Paul N, Halary S, Gros F, Fouillen L, Haeberle AM, Royer C, Spiegelhalter C, André-Grégoire G, Mittelheisser V, Detappe A, Murphy K, Timpson P, Carapito R, Blot-Chabaud M, Gavard J, Carapito C, Vitale N, Lefebvre O, Goetz JG, Hyenne V (2021). Ral GTPases promote breast cancer metastasis by controlling biogenesis and organ targeting of exosomes. Elife.

[100] Batrakova EV, Kim MS (2015). Using exosomes, naturally-equipped nanocarriers, for drug delivery. J Control Release.

[101] Akoto T, Saini S (2021). Role of exosomes in prostate cancer metastasis. Int J Mol Sci.

